# Disease-Specific Enteric Microbiome Dysbiosis in Inflammatory Bowel Disease

**DOI:** 10.3389/fmed.2018.00304

**Published:** 2018-11-20

**Authors:** Hengameh Chloé Mirsepasi-Lauridsen, Katleen Vrankx, Jørgen Engberg, Alice Friis-Møller, Jørn Brynskov, Inge Nordgaard-Lassen, Andreas Munk Petersen, Karen Angeliki Krogfelt

**Affiliations:** ^1^Department of Bacteria, Parasites and Fungi, Statens Serum Institut, Copenhagen, Denmark; ^2^Applied Maths NV, Sint-Martens-Latem, Belgium; ^3^Department of Clinical Microbiology, Slagelse Hospital, Slagelse, Denmark; ^4^Department of Clinical Microbiology, Hvidovre University Hospital, Hvidovre, Denmark; ^5^Department of Gastroenterology, Herlev University Hospital, Herlev, Denmark; ^6^Department of Gastroenterology, Hvidovre University Hospital, Hvidovre, Denmark

**Keywords:** inflammatory bowel disease, ulcerative colitis, crohn's disease, intestinal microbiome, ileo-anal pouch, dysbiosis, shannon diversity index, pielou index

## Abstract

Inflammatory Bowel disease (IBD) is traditionally divided into Crohn's disease (CD) and ulcerative colitis (UC). UC is a relapsing non-transmural inflammatory disease that is restricted to the colon and is characterized by flare-ups of bloody diarrhea. CD is a chronic, segmental localized granulomatous disease that can affect any part of the entire gastrointestinal tract. Ileo-anal pouch is a procedure restoring functionality of the rectum after a colectomy. IBD is a multifactorial disease and flares of IBD are probably triggered by changes in the intestinal microbiota followed by an abnormal immune response. In this study, we aim to analyze the intestinal bacterial diversity in IBD patients during various stages of disease compared with healthy controls. Permission for human experiments and recruitment of participants was obtained from the Ethic Committee for Copenhagen County hospitals (Permission no. KA-03019, Permission no. KA-20060159). Stools from 26 healthy controls, 42 CD, 38 UC and 18 pouch patients were collected. Stool DNA extraction was performed using Qiagen, DNA mini stool kit Denmark. DGGE-PCR amplifying the V2-V3 region of 16S-rDNA gene of the bacteria was amplified by universal primers HDA1 and HDA2. Analysis of DGGE was performed blinded using BioNumerics version 7.5. After normalization, a DGGE gel band matching was performed. The similarities between profiles were calculated with a ranked Pearson correlation coefficient based on the band matching results using band intensities. Simpson's index of diversity and Pielou's species evenness were calculated. Based on the Shannon Diversity Index, UC patients had lower species diversity and bacterial evenness in comparison to healthy persons, *p* < 0.05. However, only CD and disease pouch patients had lower species diversity compared to those with inactive disease and healthy controls. Well-functioning pouch patients had decreased species evenness in comparison to diseased pouch patients and control group. During the active disease stage in CD and pouch, the patients have a low bacterial diversity in their gut when compared to the inactive stage. In UC patients, a generally low diversity was observed at all stages of the disease compared to healthy controls.

## Introduction

Crohn's disease (CD) and ulcerative colitis (UC) are the two main forms of inflammatory bowel disease (IBD), characterized by periods of remission and relapses (1). CD is a chronic inflammatory condition that involves any part of the gastrointestinal tract, while UC is a chronic inflammation localized in the colon (1). The etiology of IBD is still unknown, but many studies indicate a combination of nutrition (2, 3), intestinal dysbacteriosis (4, 5), and abnormal immune response (6–8). During the last two decades, the focus has been on the role of microbiota as a driver of the inflammatory process in IBD.

One theory suggests that living in an industrialized country with high hygiene standards lowers the exposure to complex microbial communities during the development of the immune system. As a result, the immune system is less able to tolerate exposure to environments rich on microbiota resulting in an inappropriate immune activation (6). Humans live in a symbiotic relationship with the gut microbiome, in which humans provide the ideal environment for the microbiota to flourish and bacteria in return ferment the food carbohydrates to short fatty acids, synthesize certain vitamins and degrade dietary oxalates (2–5).

Studies show that IBD with active disease is strongly associated with an overall increased prevalence of proteobacteria (9, 10) and drop in intestinal species richness, more specifically a significant drop in firmicutes (11, 12), *Enterobacteriaceae, Bacteroidales*, and *Clostridiales* (13) of Clades IV and XIVa (13). Decreased prevalence of butyrate-producing bacteria such as *Clostridiales* species in the gut explains the decreased amount of short chain fatty acids in fecal samples from IBD patients (14, 15). Butyrate serves as a major source of energy for colonic epithelial cells (16) and as an inhibitor of pro-inflammatory cytokine expression in the intestinal mucosa through a mechanism that involves hyper-acetylation of histones and suppression of NF-κB signaling (17). Butyrate reinforces the mucosal barrier by inducing production of mucin and antimicrobial peptides, and by strengthening epithelial barrier integrity through directly increasing the expression of tight junction proteins (18). More recent studies indicate a link between IBD and a decreased prevalence of butyrate-producing *Faecalibacterium prausnitzii* (firmicute), belonging to Clostridia cluster IV species (19, 20), increased prevalence of adherent-invasive *E. coli* (AIEC) in ileal CD (21, 22) and increased prevalence of *E. coli* with extra intestinal pathogenic *E. coli* properties (23–25). It has been shown that fecal microbiota transplantation (FMT) is an effective treatment of UC, indicating that colon microbiota play an essential role in UC (26). A subdivision of IBD into three phenotypes as ileal CD, colonic CD, and UC according to genetic composition has been suggested (27).

In this study, the goal was to investigate intestinal bacterial diversity in IBD patients with active and inactive disease compared to healthy controls, using PCR amplification of regions of the 16S ribosomal RNA gene and Denaturing Gradient Gel Electrophoresis (DGGE).

## Material and methods

This study included 97 adult IBD patients with a previously confirmed IBD diagnosis and 31 healthy controls, aged from 25 to 86 and 18 to 67 years, respectively. Symptom scores, stool and blood samples were collected from 38 patients with UC, 15 females and 23 males, 20 with active (UCA) and 18 with inactive (UCI) disease, 41 patients with Crohn's disease, 28 females and 13 males, 21 with active (CDA) and 20 with inactive (CDI) disease, 18 patients with an ileo-anal pouch (UC patients who underwent surgical treatment), 8 females and 10 males, including 7 pouchitis (PA), and 11 well-functioning pouch (PI) patients. The disease activity was measured with the Simple Clinical Colitis Activity Index (SCCAI) (28), the Harvey-Bradshaw Index (HBI) (29), and the Modified Pouchitis Disease Activity Index (MPDAI) (30). All the participants answered the questionnaires regarding medicine or antibiotic usage, travel diarrhea/diarrhea in the last 3 months before participating in the study. Patients or controls, who used antibiotics or corticoides 3 months before the start of the study, were excluded. Overall, 98% of the study group originated from Denmark, while the last 2% were integrated Danish citizens of other ethnicity.

The stool samples were stored at −80°C and thawed before processing. The laboratory staff was blinded to patient histories.

### Ethics statement

Permission for the study was obtained from the Regional Ethics Committee for Copenhagen County Hospitals (Permission no. KA-03019, Permission no. KA-20060159) and all participants gave their informed written consent. Healthy controls were recruited among students and volunteers. Patients and healthy controls completed a questionnaire about their condition and their medication. IBD patients were diagnosed according to standardized criteria (29, 31).

### DNA extraction

DNA extraction was performed according to the instructions of the manufacturer (Qiagen, DNA stool mini kit Denmark) with the following modifications: 100 mg fecal sample was mixed with 1.4 ml ASL buffer in a 2-ml tube and vortexed until the sample was thoroughly homogenized. Samples subsequently mixed with 0.2 g sterile zirconia/silica beads. Hereafter, the samples were processed on a TissueLyser for 6 min at 30 Hz. Lysis was completed at a temperature of 95° C for 5 min. Finally, DNA was extracted according to the instruction of the QIAamp DNA stool MiniKit and eluted in 100 μl elution buffer provided in the kit.

### PCR amplification

The V2-V3 region of the 16S rDNA gene (~200 bp) was amplified using the universal primer set HDA 1 position 338-357: (5′ACT CCT ACG GGA GGC AGC AGT′3) and HDA 2 position 539–561: (5′GTA TTA CCG CGG CTG CTG GCA C–′3) (32). The forward primer, HDA 1, was at the 5′end labeled with GC clamp (5′CGC CCG GGG CGC GCC CCG GGC GGG GCG GGG GCA CGGGGG G ′3). PCRs were performed in a total volume of 50 μL containing 20 μL of 5 PRIME Mastermix, 0.8 μM primer HDA 1-GC, 0.8 μM primer HDA 2, 10 μL of DNA template and, finally, 4 μL RNase free water.

The PCR was performed using the following conditions: preheating at 94°C for 4 min proceeded by 30 cycles of denaturing at 94°C for 30 s, annealing at 56°C for 30 s, elongation at 68°C for 45 s, and finally a single step of 68°C for 7 min. The PCR products were run on a 0.8% agarose gel.

### Denaturing gradient gel electrophoresis

PCR fragments were separated by Denaturing Gradient Gel Electrophoresis (DGGE) with DCode System according to the manufacturer's instructions. Eight percent Polyacrylamide (vol/vol) [ratio of acrylamid:bisacrylamide (37.5:1)] was diluted in 0.5xTAE buffer with pH 8.0 using a gradient ranging from 35 to 65% [100% acrylamide corresponds to 7 M urea and 40% (vol/vol) formamide] (33). Gels were cast using a gradient maker and a pump with a flow speed of 5 mL per minute. After polymerization of the gel (2 h), a 3% stacking gel without denaturing chemicals was cast, and an appropriate comb was subsequently inserted and left for 30 min for polymerization. Gels were run at 60°C for 16 h at a constant voltage of 70 V in 0.5x TAE buffer. After electrophoresis, gels were stained with GelRED for 45 min and analyzed using an ultraviolet trans-illuminator.

### Data analyses

Analysis of the DGGE was performed blinded using BioNumerics version 7.5 (Applied Maths NV, Sint-Martens-Latem, Belgium). After normalization, a band matching was performed with a tolerance of 1% and an optimization of 1%. The results from the automatic band matching were checked manually and corrected where necessary. The similarity between profiles was calculated with a Pearson correlation. A dendrogram was then constructed with UPGMA. The reliability of the dendrogram was determined with a cophenetic correlation coefficient. This coefficient compares a similarity matrix derived from the dendrogram with the actual similarity matrix. Reliably separated branches have a high cophenetic correlation. For each profile, the Shannon Diversity Index of diversity and Pielou's species evenness were calculated. Linear discriminant analysis (LDA) was used to analyze differences within patient groups and healthy persons. All statistical analyses were performed after mean-based normalization using analysis of variance (Anova) with *post hoc* test Bonferroni. The level of significance was set at *p* ≤ 0.05.

## Results

**Figure 1:**
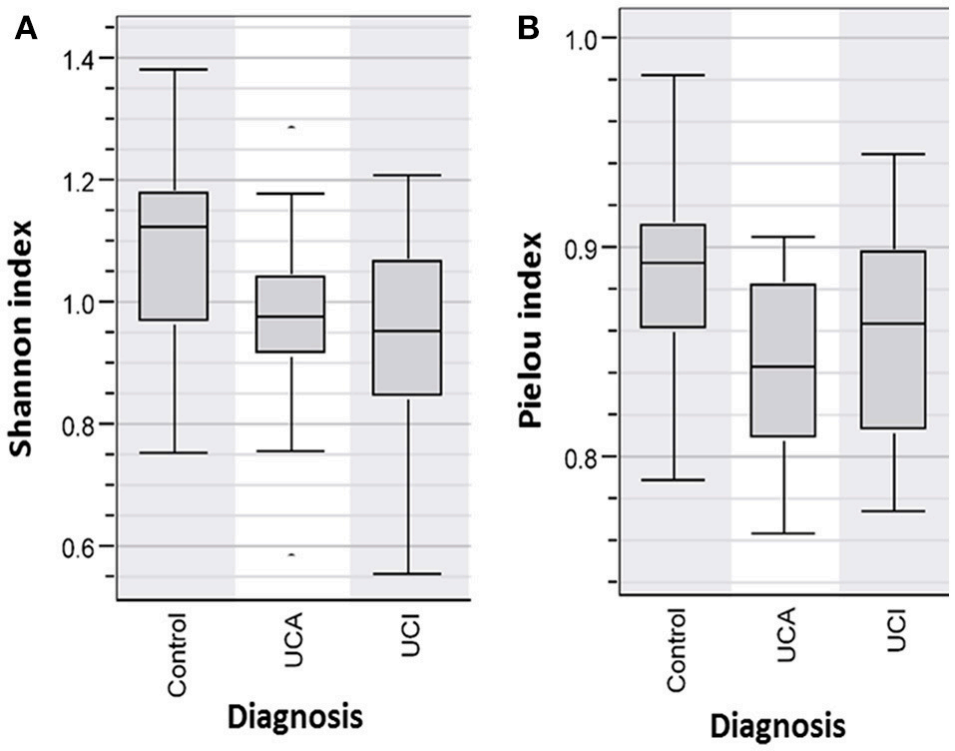
**(A)** Shannon Diversity Index for patients with active (UCA) and inactive (UCI) Ulcerative colitis vs. healthy person/Control group shows patients group (UCI and UCA) have a significantly decreased bacterial abundance in comparison to controls group, *p* < 0.05^*^(B) Pielou index shows significantly decreased bacterial evenness in the patient groups (UCI and UCA) vs. controls, *p* < 0.05.

DNA was extracted from fecal samples and analyzed by DGGE. The individual bands each representing a bacterium were analyzed in BioNumerics. The microbiota was correlated to the diagnosis. Based on the Shannon Diversity Index, UC patients with active and inactive disease had significant lower bacterial diversity in comparison to healthy controls, *p* < 0.05^*^ (Figure 1A). When comparing the species evenness using Pielou's index, UC patients had decreased species evenness compared with healthy controls, *p* ≤ 0.05 (Figure 1B). No such difference was found between UC patients with active and inactive disease, respectively.

**Figure 2:**
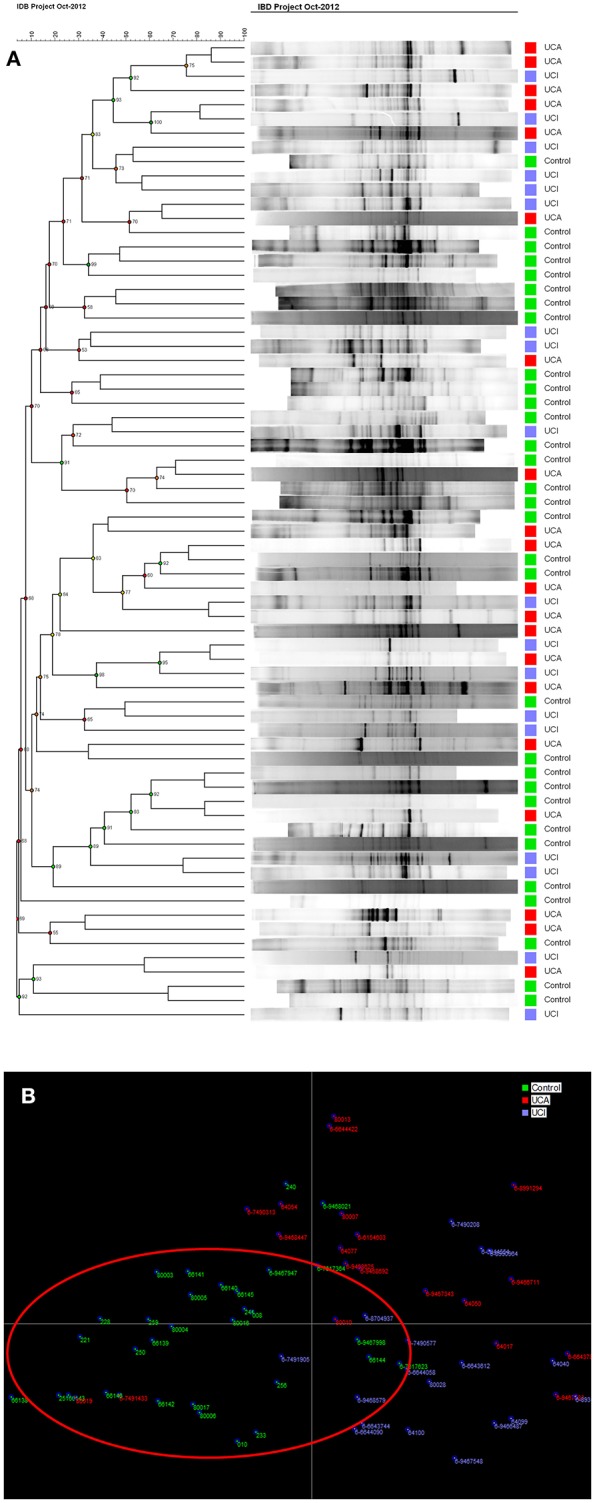
**(A)** Cluster analysis of control group and patient groups with active (UCA in red)/inactive (UCI in blue) ulcerative colitis. **(B)** LDA shows that controls in green are mostly gathered in the red circle.

Not only a reduced number of bands (low bacterial diversity), but also brighter bands (increased bacterial concentration), were observed using DGGE profiles in UC patients compared to healthy controls. When using cluster analysis (Figure 2A), UC patients with (red) and without (blue) active disease were separated from healthy controls (green). This was confirmed by linear discriminant analysis (LDA; Figure 2B), where the controls in the red circle were separated from patients (Figures 2A,B).

**Figure 3:**
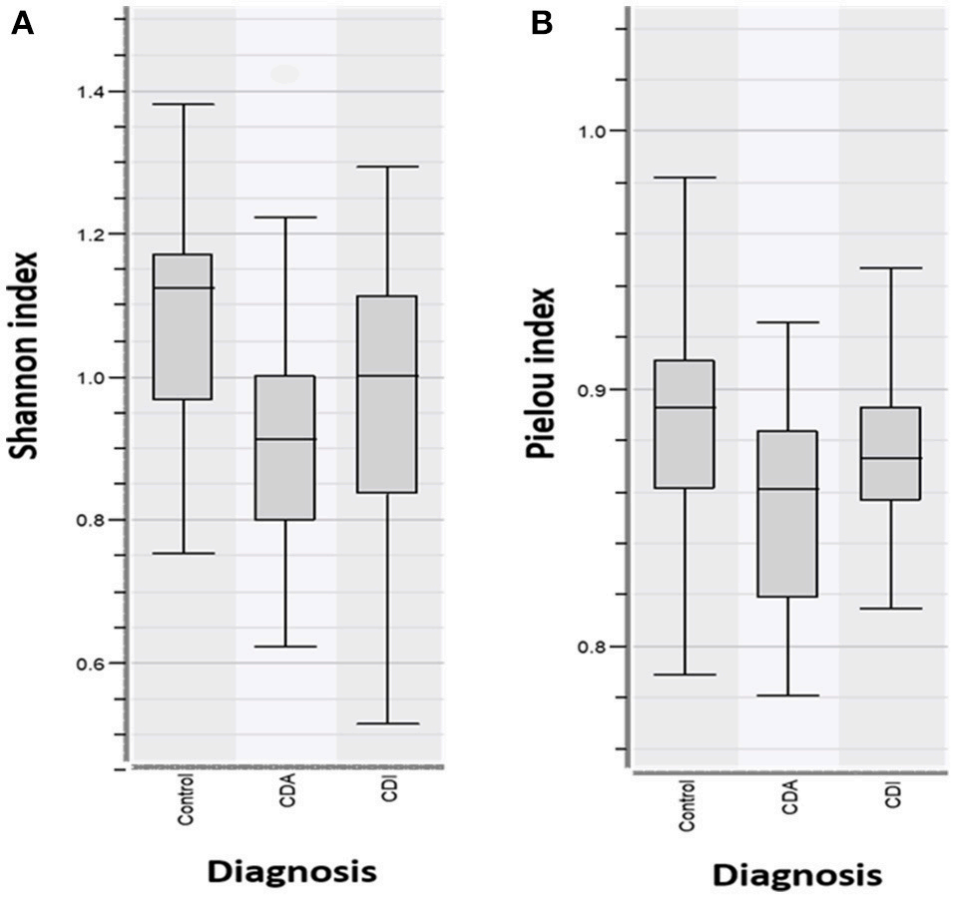
**(A)** Shannon Diversity Index for CD patients with active (CDA) and inactive disease (CDI) vs. controls show CDA have significantly decreased bacterial abundance in comparison to CDI and controls, *p* > 0.05*. **(B)** Pielou index shows no significant differences in species evenness in patient groups vs. controls (*p* < 0.083).

Analysis of the bacterial diversity of CD patients with active (CDA) and inactive (CDI) disease in comparison with controls showed that CD patients with active disease had a significantly lower Shannon Diversity Index in comparison to healthy persons, *p* < 0.05^*^ (Figure 3A). When comparing the species evenness using Pielou's index, no significant differences were found between patient groups vs. control group/healthy persons, *p* < 0.083 (Figure 3B).

**Figure 4:**
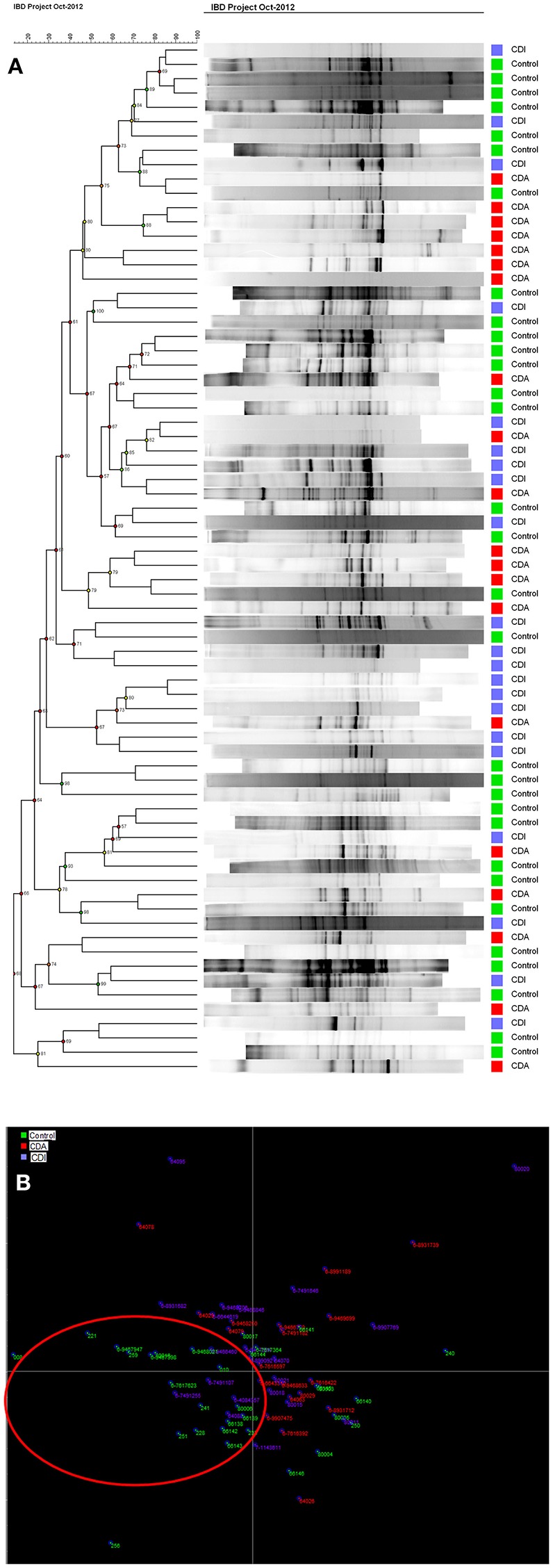
**(A)** Cluster Analysis of controls and patient groups with active (CDA, in red) and inactive (CDI, in blue) Crohn's disease shows the group clustering in colors **(B)** LDA shows control in green are mostly separated in the red ring, from CDA in red and CDI in blue.

Cluster analysis (Figure 4A) showed that generally profiles from CD patients with (CDA, red) active disease could be found in the same cluster as other CDA profiles. The same was seen for CDI and control group. However, many exceptions were found and the clusters were not clearly delineated. This effect was seen more clearly with LDA, where healthy controls (in green) shown in the red circle, were separated from the patients (Figure 4B). No separation could be observed in the CDA and CDI analysis.

**Figure 5:**
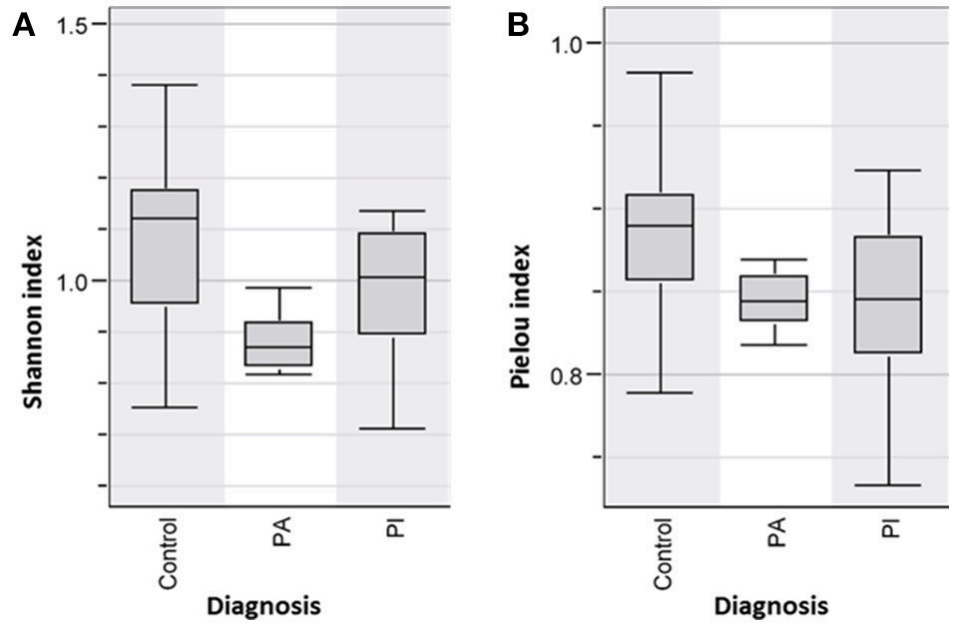
**(A)** Shannon Diversity Index of pouch patient groups with active (PA) and inactive disease (PI) and controls shows PA has significant decreased bacterial diversity index in comparison to the PI and control group, *p* < 0.05. The differences were not significant in PI, *p*-value = 0.06. **(B)** Species evenness (Pielou index) was lower in the patient groups compared to the healthy controls, however these differences were only significant in PI, *p* < 0.05.

In comparison to healthy persons and well-functioning pouch patients (PI), diseased pouch patients (PA) had a significantly decreased Shannon Diversity Index, *p* < 0.05 (Figure 5A). When comparing the species evenness using Pielou's evenness index, a lower evenness was found in the patient groups; however, only the difference between controls and PI reached significance *p* < 0.05 (Figure 5B).

**Figure 6:**
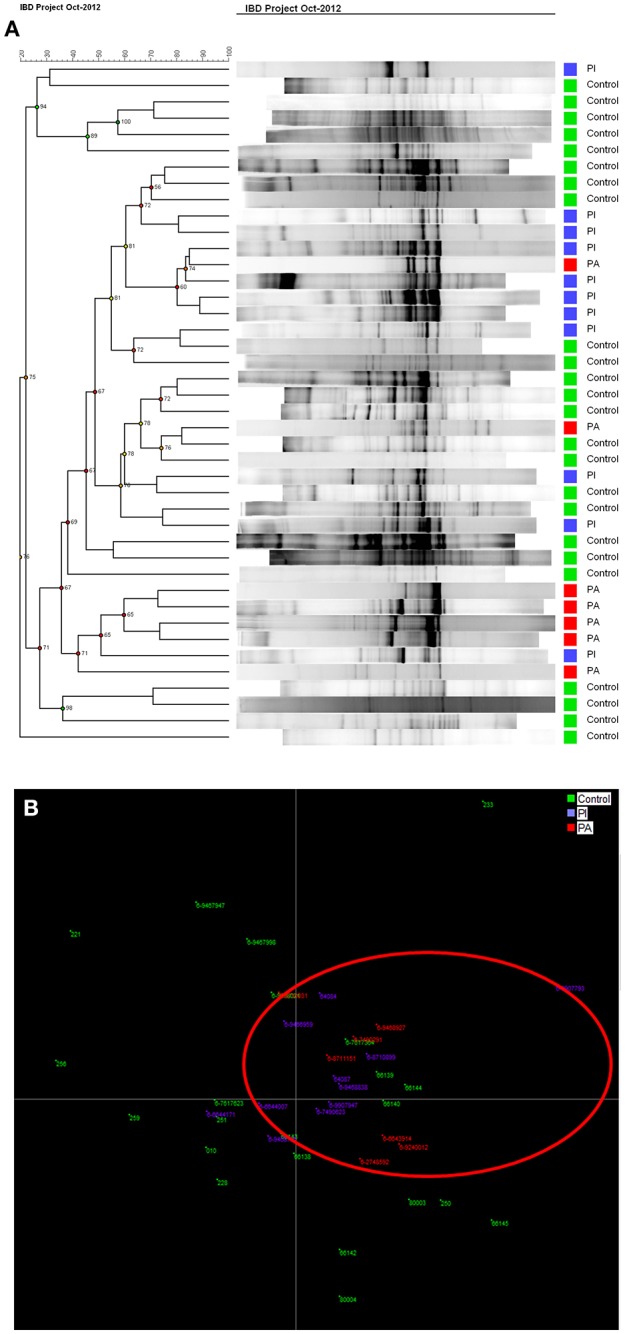
**(A)** Cluster analysis for pouch patients with active (PA, in red) and inactive (PI, in blue) disease shows controls (green) clustered mostly together. **(B)** LDA graph shows most of controls in green are separated from PI in blue and PA in red.

Cluster analysis (Figure 6A) showed that DGGE profiles from diseased pouch patients (red) and well-functioning pouch patients (blue) were more similar to profiles from other patients, while healthy controls also clustered with other healthy controls (green). However, the clusters were not clearly delineated. A better separation could be seen by LDA, where the majority of controls separated from the patients shown in red circle (Figures 6A,B).

## Discussion

The pathogenesis of IBD is still not clear. However, during the last decades, the main focus has been on an imbalance in the intestinal microbiome in IBD patients that might cause or trigger the disease activity. Next generation sequencing investigation of the gut microbiome provides large amounts of data, which can be difficult to interpret, and it is still quite costly. The advantages of using DGGE (34) compared with next generation sequencing is that DGGE detects highly prevalent (high concentration) intestinal bacteria, while next generation sequencing is random and detects all DNA. Sequencing is biased by the primer annealing, and even small amounts of DNA will be amplified. In this study, we investigated the intestinal microbiota in IBD using 16S rDNA PCR and DGGE enabling the discovery of overall differences in intestinal bacteria community within different IBD groups and controls. When discussing the concept of diversity of a microbial community, the terms species evenness and richness are used. Bacterial richness, also known as the Shannon diversity index is a measurement of the total number of species in the community, while evenness expresses how evenly the individuals in the community are distributed over the different species (35). As the colon contains two-thirds of the total amount of bacteria in the human body (36), it is an exceptional environment, and a perfect symbiosis plays a key role in colon health. UC is localized in the colon and is linked to intestinal bacterial dysbiosis and reduced biodiversity (37). This is also confirmed in our study, where UC patients with and without active disease had significantly reduced Shannon diversity indices and reduced fecal bacterial evenness in comparison with healthy persons (Figure 1). LDA and cluster analysis (Figure 2B) confirm this result, showing a separation between UC patients and healthy controls. Yet, the question of what triggers the disease activity in UC patients remains. If bacteria in the colon are responsible, what are the differences in the intestinal bacteria community in the UC patients with and without active disease? As we see an overall reduced biodiversity in UC patients, the flares of UC might be triggered by the shift between the different bacterial communities in UC patient. The studies using next generation sequencing reveal that a reduced amount of *F. praustnitzii* (38) and an increased number of Proteobacteriae (39, 40) might have an effect on UC relapses. In order to restore the biodiversity, fecal microbiota transplantation (FMT) was performed and it was shown to be effective to promote remission in UC patients with active disease, confirming the role of the colon microbiota in UC (26).

CD is a chronic inflammatory condition that involves any part of the gastrointestinal tract. Colonic CD has been identified in 60% of the CD patients (41), which often present with diarrhea and an aggressive course of the disease. Intestinal dysbiosis is also linked to colonic CD, with increased prevalence of *Faecalibacterium* (Firmicutes) and two unidentified genera of Clostridiales and Ruminococceae (42), while ileal patients have a higher prevalence of Proteobacteria compared to healthy subjects. Our results show that CD patients with active disease have a significantly reduced Shannon diversity index in comparison with CD patients with inactive disease and healthy controls (Figure 3A). Decreased species evenness was found in CD patients with and without active disease compared to healthy persons; however, these differences were not significant, *p* = 0.08 (Figure 3B). Interestingly, a metanalysis performed on FMT in CD, reveals that FMT promotes remission in only 60% of the CD patients (43), as only 60% of the CD patients had colonic CD (41). Reduced bacterial diversity in children with CD was reported previously (40, 44, 45).

Ileal pouch-anal anastomosis is a surgical treatment choice for the majority of UC patients, who fail medical treatment therapy, with the advantage of re-establishment of gastrointestinal continuity and improvement of the quality of life for these patients. Studies show that 40% of patients with ileal pouch-anal anastomosis develop pouchitis during the first year after surgery (46). Furthermore, antibiotic and probiotic treatment appears to be effective to diminish pouchitis (47). Evidence suggests that an abnormal mucosal immune response to altered microbiota in the pouch leads to acute chronic inflammation (48). Our analysis of bacterial diversity in pouch patients in comparison with healthy persons shows that diseased pouch patients (PA) have a significant decreased microbial diversity in comparison to well-functioning pouch patients and healthy persons, *p* < 0.05 (Figure 5A). Reduced diversity was shown in well-functioning pouch patients in comparison to healthy controls; however, the differences are not significant (Figure 5A). Additionally, our results reveal significantly decreased fecal bacterial evenness in well-functioning pouch patients suggesting that the microbiota in these patients is dominated by just a few species. Maharshak et al. (49) show that pre-pouchititis patients have significantly decreased intestinal bacterial diversity 1 year before pouchitis in comparison to pouch patients, who did not develop pouchitis. The possibility of subclinical inflammation 1 year before pouchitis was confirmed using histological scores, endoscopics and fecal calprotectin (49). This study supports our results showing that well-functioning pouch patients have significantly decreased fecal bacterial evenness and reduced Shannon diversity indices in comparison to healthy persons, which was also confirmed by our LDA analysis (Figure 6B). It could be speculated whether most of the well-functioning pouch patients in our cohort are in the stage of pre-pouchitis, shown by reduced intestinal bacteria evenness in these patients. Increased relapses in pouch patients might be explained by the surgical treatment where the pouch has a “colon like” function, which creates a good environment for bacteria to overgrow and cause infections. This supports the finding that FMT does not promote remission in pouchitis patients (50) as antibiotics do.

Microbial colonization has an important effect on the instruction and regulation of the immune system (51). Abnormal communication between gut microbial communities and the mucosal immune system has been identified as the core defect that leads to chronic intestinal inflammation (52). Current studies are focusing on restoring a healthy intestinal microbiota using FMT and probiotics to treat the intestinal bacterial dysbiosis. This might open new treatment opportunities for IBD patients treated with immune suppressive medication or for those where medical treatment therapy fails.

## Conclusion

This study shows that UC patients with and without active disease have significantly decreased intestinal bacterial diversity and evenness in comparison to healthy persons. However, CD patients and diseased pouch patients had significantly decreased intestinal bacterial diversity, during the active disease compared to healthy persons, CD patients and well-functioning pouch patients without active disease.

The well-functioning pouch patients had significantly decreased species evenness in comparison to diseased pouch patients and healthy persons.

## Author contributions

AP, KK, JE, AF-M, IN-L, HM-L, and JB participated in the design of the study. KV, AP, KK, and HM-L drafted the manuscript. HM-L and KV were responsible for the experimental setting. All authors have read and approved the final manuscript.

### Conflict of interest statement

The authors declare that the research was conducted in the absence of any commercial or financial relationships that could be construed as a potential conflict of interest.

## Abbreviations

IBD: Inflammatory Bowel Disease
UC: Ulcerative colitis
UCA: Ulcerative colitis with active disease
UCI: Ulcerative colitis with inactive disease
CD: Crohn's disease
CDA: Crohn's with active disease
CDI: Crohn's with inactive disease
PA: Disease pouch
PI: well-functioning pouch.
